# Identifying socio-demographic and socioeconomic determinants of health inequalities in a diverse London community: the South East London Community Health (SELCoH) study

**DOI:** 10.1186/1471-2458-11-861

**Published:** 2011-11-11

**Authors:** Stephani L Hatch, Souci Frissa, Maria Verdecchia, Robert Stewart, Nicola T Fear, Abraham Reichenberg, Craig Morgan, Bwalya Kankulu, Jennifer Clark, Billy Gazard, Robert Medcalf, Matthew Hotopf

**Affiliations:** 1 King's College London, Psychological Medicine, Institute of Psychiatry, 10 Cutcombe Road, London SE5 9RJ, UK; 2 King's College London, Section of Epidemiology, Health Service and Population Research Department, Institute of Psychiatry, Box P060 De Crespigny Park London SE5 8AF, UK; 3 King's College London, Academic Centre for Defence Mental Health, 10 Cutcombe Road, London SE5 9RJ, UK; 4 King's College London, Section of Social Psychiatry, Health Service and Population Research Department, Institute of Psychiatry, Box P063 De Crespigny Park London SE5 8AF, UK

## Abstract

**Background:**

Responses to public health need require information on the distribution of mental and physical ill health by demographic and socioeconomic factors at the local community level.

**Methods:**

The South East London Community Health (SELCoH) study is a community psychiatric and physical morbidity survey. Trained interviewers conducted face-to-face computer assisted interviews with 1698 adults aged 16 years and over, from 1076 randomly selected private households in two south London boroughs. We compared the prevalence of common mental disorders, hazardous alcohol use, long standing illness and general physical health by demographic and socioeconomic indicators. Unadjusted and models adjusted for demographic and socioeconomic indicators are presented for all logistic regression models.

**Results:**

Of those in the sample, 24.2% reported common mental disorder and 44.9% reported having a long standing illness, with 15.7% reporting hazardous alcohol consumption and 19.2% rating their health as fair or poor. The pattern of indicators identifying health inequalities for common mental disorder, poor general health and having a long term illness is similar; individuals who are socioeconomically disadvantaged have poorer health and physical health worsens as age increases for all groups. The prevalence of poor health outcomes by ethnic group suggests that there are important differences between groups, particularly for common mental disorder and poor general health. Higher socioeconomic status was protective for common mental disorder, fair or poor health and long standing illness, but those with higher socioeconomic status reported higher levels of hazardous alcohol use. The proportion of participants who met the criteria for common mental disorder with co-occurring functional limitations was similar or greater to those with poor physical health.

**Conclusions:**

Health service providers and policy makers should prioritise high risk, socially defined groups in combating inequalities in individual and co-occurring poor mental and physical problems. In population terms, poor mental health has a similar or greater burden on functional impairment than long term conditions and perceived health.

## Background

Even before the birth of modern epidemiology, inner cities have been recognised to represent particular challenges to individual health and public health [1,2]. Densely populated areas allowed spread of infectious disease; sanitation was often poor and air pollution was, and continues to be, a health hazard. Modern inner cities have pockets of extreme poverty and overcrowding, sometimes juxtaposed with areas of considerable affluence. More than half of the world's population resides in modern inner cities [1], most of which can be characterised by inequalities in education, employment opportunities, affordable and safe housing, location of toxic environments, and availability of affordable nutritious food supplies [2,3]. These are the social inequalities that beget health inequalities [3].

Whilst the nature of health hazards associated with urban living have changed, health inequalities associated with living in inner cities remain an issue of considerable concern. The pattern is complex - in the UK, some inner city areas (e.g. Kensington and Chelsea, London) have among the highest life expectancies; whilst others (particularly in Scotland and northern England) have the lowest [4]. Within London, there are considerable differences in life expectancy according to location, with variations of greater than six years over fewer miles [4]. Less is known about morbidity in high risk populations at the local community level, particularly in relation to the prevalence of psychiatric disorders and long term health conditions.

In the UK, inner cities are also likely to be the first port of call for migrants and as with migrants to other cities, many with general health problems influenced by former circumstances, such as exposure to conflict and war, persecution or poverty [5,6]. At the same time, many migrants to industrialised cities will have come to work or study and represent a relatively healthy constituency of the population. The history of inner London has seen wave upon wave of such migration from the (protestant) Huguenots of France or the Jewish communities of middle Europe escaping religious persecution in the 17^th^-20^th ^centuries, to economic migration from former British colonies and more recently Eastern Europe in the 20^th ^and 21^st ^century, and people now seeking asylum from conflict zones in the Middle East, North Africa and Central Asia. Migration patterns in London and other cities worldwide result in continuous demographic and socioeconomic transformations and potential shifts in health profiles of these groups. Thus, the generation of data at the local community level should be an ongoing, integrative process that allows for the identification of high risk groups as an initial step towards understanding how the experience of demographic and socioeconomic statuses become part of a complex matrix of exposures implicated in poor mental and physical health.

A wide range of mental and physical health indicators are needed to begin to identify and understand health inequalities within local communities. Common mental disorder as a term has been used frequently to describe the impact of mental disorders in the community, recognising the fact that distinct diagnostic constructs such as anxiety and depression used in secondary care do not adequately represent the mixed symptomatology that is more often seen in epidemiological samples [7]. For example, the predominating syndrome in British national surveys of psychiatric morbidity in community residents has been mixed anxiety and depressive disorder, far outweighing in prevalence and impact those fulfilling conventional diagnostic criteria for depressive or specific anxiety disorders [8-10]. Common mental disorder has often been found to have a higher prevalence than physical health problems [11] and is more prevalent in residents of urban environments [12]. Common mental disorder, often untreated, is a major public health challenge in part due to it being a significant source of impairment and poor social functioning [7]. Effective mental health care has also been identified as a prerequisite for good general health [13].

Substance use, alcohol being among the most commonly used, has also been a focus of building health profiles due to its association with morbidity, mortality and high social, medical, and economic costs [14]. However, the evidence related to identifying the social distribution of alcohol use is mixed [15]. For example, both higher and lower socioeconomic status groups have been associated with a greater likelihood of alcohol use and dependence in studies on national samples, whereas other studies have shown no difference [16-19].

Along with capturing longstanding illness and functional limitations, self-rated health is a valuable source of subjective health status and an important indicator of overall health, as well as a significant predictor of morbidity and mortality [20,21]. Self-rated health is associated with socioeconomic status, as measured by educational attainment, income level and social class [22-24] and recent evidence from the UK shows that it decreases with age in both men and women [24].

The majority of information about the distribution of mental and physical ill health by demographic and socioeconomic characteristics has come from national studies, such as the National Comorbidity Study in the US (e.g., [25]) and the Office for National Statistics psychiatric morbidity surveys in the UK (e.g., [18]). National surveys usually do not allow for local analysis due the lack of adequate sampling at the local level, restricted inclusion of local areas, and methodological challenges related to inferring estimates from national data [26]. In addition, the local differences by key demographic and socioeconomic indicators, such as ethnicity and levels of deprivation make local analysis necessary for developing accurate public health strategies. With growing emphasis on the need for health research to be translational (i.e., of benefit to improving treatments and/or quality of patient care), there is an even stronger need for locally relevant epidemiological evidence that serves to identify mental and physical public health needs in the population from which clinical populations are drawn [27].

In response to this need, we conducted a community based epidemiological study, developed by and in partnership with the clinicians serving the local population, to provide relevant prevalence estimates of mental and physical health symptoms in an ethnically and socioeconomically diverse, geographically defined, inner city community. This paper addresses the following aims: (1) to estimate the prevalence common mental disorders, hazardous alcohol consumption, general physical health and the presence of a long standing illness; (2) to investigate (a) the distribution of health outcomes by socio-demographic and socioeconomic indicators and (b) associations between health outcomes; and (3) to examine the association between the health outcomes of interest and functioning in work or other daily activities. The outcomes were chosen to capture morbidity of general mental and physical ill health and when present, have been identified as determinants of functional limitations. Thus, we selected available measures that represent mood and anxiety disorders and substance misuse, self-rated health and chronic physical illness.

## Methods

### Study design and participants

The South East London Community Health (SELCoH) study is a community survey of psychiatric and physical morbidity of 1698 adults, aged 16 years and over from 1075 randomly selected households in the south London boroughs of Southwark and Lambeth. In the two boroughs, there is higher deprivation than the England average, but similar proportions of economically active and inactive residents in comparison to greater London [28-31]. The boroughs are also ethnically diverse, with a greater number of Black Caribbean residents but fewer South Asian residents than other areas of London [32]. The SELCoH sample resided in a community setting served by South London and Maudsley National Health Service Foundation Trust (SLaM), and the partnership between King's College London and SLaM allows this and other research to inform and benefit clinical treatment.

From 2008-2010, households (defined as one person or group of people who have the accommodation as their only or main residence and for groups who either share at least one meal a day or share the living area) were identified through random sampling, applying similar methods to the British National Psychiatric Morbidity surveys [33] - i.e. retrieving addresses from the Small User Postcode Address File (PAF), which has near complete coverage of private households in the UK. The PAF excludes postcodes that receive more than 50 items of mail per day (which are likely to be public institutions and businesses). It should be noted that some addresses that were non-residential (i.e. businesses), shared (i.e. sheltered accommodation, student housing), or vacant (i.e. no tenants or being-demolished) were selected. These addresses were excluded once they were visited and confirmed as non-private households. The sample was stratified across the two boroughs to ensure a similar sample size for each area.

### Procedures

Having sent a letter describing the study two weeks in advance, interviewers visited each selected household at least four times at different times of day, and on weekdays and weekends, before closing the household from selection due to non-response. During each household visit interviewers attempted contact with a resident to describe the study, inform them that participation was voluntary, seek consent and conduct as many interviews or make as many appointments for interviews as possible.

We designed a computer assisted interview schedule and carried out a pilot study to assess reliability, validity and feasibility of the study procedures and questionnaire. Closely supervised, trained interviewers conducted face-to-face interviews with these schedules. The survey questionnaire collected information on the following topics: (1) socio-demographics; (2) migration; (3) socioeconomic status (SES); (4) psychosocial factors (e.g., social support); (5) neighbourhood characteristics; (6) social adversity; (7) health behaviours; (8) physical and mental health symptoms; and (9) treatment and health service use. Translators were used in interviews with non-English speaking adults. Participants received 15 GBP for a completed interview. The study received approval from the King's College London research ethics committee, reference CREC/07/08-152.

### Measures

#### Health outcomes

Common mental disorder was assessed with the Revised Clinical Interview Schedule (CIS-R)[34]- a structured interview that asks about the following 14 symptom domains (using skips to allow asymptomatic individuals to answer a minimum of 28 questions): fatigue, sleep problems, irritability, worry, depression, depressive ideas, anxiety, obsessions, subjective memory and concentration, somatic symptoms, compulsions, phobias, physical health worries and panic. A total CIS-R score at or above 12 is conventionally used to indicate the presence of common mental disorder (CMD). The CIS-R also provides ICD-10 diagnoses for ten mental disorders through a standard algorithm.

Hazardous alcohol use was measured with the Alcohol Use Disorders Identification Test (AUDIT) [35], developed by the World Health Organization (WHO). The measure comprises ten questions relating to alcohol consumption, symptoms of alcohol dependence and problems related to alcohol abuse within the last 12 months. Each item is scored 0-4 with a summed overall score ranging from 0-40. An AUDIT score of 8 or more has been used to define hazardous alcohol use [35].

Fair or poor general health was indicated by self-rated current general health, a single item from the 12-item Short Form (SF-12) questionnaire [36]. Individual items from the SF-12 were also used as functioning indicators. Limitations in social functioning were indicated by participants reporting how much time in the past four weeks their physical health or emotional problems interfered with their social activities (e.g., visiting friends or relatives). The response categories were none, some or most of the time. Functional limitations due to physical health represented participants who indicated that their physical health limited the kind of work or other activities they could do during the past 4 weeks (1 = quite a bit). Functional limitations due to emotional health was defined by participants indicating they did not do work or other activities as carefully as usual in the past four weeks due to their emotional health (1 = quite a bit).

Participants were classified as having a long standing illness if they indicated that they had a long-standing illness, disability or infirmity that troubled the participant over a period of time.

#### Socio-demographic indicators

Distributions of the outcomes were described by gender, ethnicity, age, relationship status and borough of residence. Self-reported ethnicity indicated identification with one of the following groups: White British, Black Caribbean, Black African, Indian, Pakistani, Bangladeshi, or Other. South Asian (i.e., Indian, Pakistani, and Bangladeshi) and Other ethnic groups were collapsed to improve distribution. A continuous age indicator was recoded into a categorical variable representing the following six groups for ease of interpretation: 16 to 24 years; 25 to 34 years; 35 to 44 years; 45 to 54 years; 55 to 64 years and 65 years and over. A participant's relationship status was classified into never married, married or cohabiting, divorced or separated, or widowed. Finally, borough indicated the location of the residence in one of the two sampled London boroughs (Southwark and Lambeth).

#### Socioeconomic indicators

Indicators of socioeconomic status included educational attainment, social class, employment status, household income and housing tenure. Educational attainment was indicated by reporting having no qualifications, qualifications up to GCSE or Ordinary level (e.g., high school diploma), qualifications up to Advanced level (e.g., advanced placement qualification) and higher degree or above (e.g., university degree). Social class was measured by current occupation categorized according to the Registrar General's classification [37] into six categories: professional (I), managerial/technical (II), skilled non-manual (III-NM), skilled manual (III-M), semi-skilled (IV) and unskilled (V). For this analysis, social class was condensed into three categories to improve the distribution and ease interpretation: (1) non-manual; (2) manual; and (3) no current occupation. The latter category was added to represent those without a current occupation needed to categorise participants in a social class group (approximately 44 percent of the sample). Employment status referred to whether or not the participant was engaging in full-time employment, part-time employment, student (either working full or part-time or not), unemployed, and economically inactive groups that include temporary sick or permanent sick/disabled, retired or looking after the home with children. Participants reported gross household income (i.e., all income sources before deductions for income tax and National Insurance) based on the following five categories: (1) £0-£5,475; (2) £5476 - £12,097; (3) £12,098 - £20,753; (4) £20,754 - £31,494; (5) £31,495 or more. To capture housing tenure, participants were asked to categorise their current accommodation in the following categories: (1) own or mortgage, (2) rented and (3) rent-free (living rent free in relative's/friend's property or squatting).

### Statistical analysis

Analyses were completed in STATA 11 [38]. We used appropriate survey commands (svy) for estimates of prevalence and associations to generate robust standard errors. All analyses of SELCoH data accounted for clustering by household inherent in the study design and weighted for non-response within households (see Pickles et al [39] for further discussion of the application of weights). We calculated inverse probability weights from the predicted response probabilities derived from a logistic regression model of whether or not an eligible household member (i.e., 16 years or older) completed the survey. Two main criteria were used in selecting effects for inclusion in the weights: 1) statistical significance within the logistic regression, and 2) the extent to which the selected weighting scheme satisfactorily reproduced the means and prevalence rates of cases with complete data. The prediction equation included effects of gender and age. We reported the unweighted frequencies for all indicators and applied the Pearson's χ^2 ^tests with Rao & Scott second-order corrections with 95 percent confidence intervals for categorical health outcomes. Odds ratios (OR) with 95 percent confidence intervals were calculated for associations between demographic and socioeconomic indicators with the categorical outcomes. Unadjusted and models adjusted for gender, age (continuous), ethnicity, relationship status, employment status, household income and housing tenure were presented for all logistic regression models.

## Results

### Sampling

Of 3600 selected addresses, 359 were declared unusable because they were not residential, not private households or vacant, 957 addresses were approached but no contact was made with household members, 31 addresses were duplicates, 16 households from the pilot study were not included in the main study and for 76 addresses, contact was made with a household member but there was no follow up contact. Thus, contact was established with 2070 private households, of which 1075 households had at least one member interviewed, representing a 51.9% household participation rate. Of 2359 people eligible within the participating households, 1698 (71.9%) participated (mean participants per household= 2.7; SD = 1.2).

As described in Table 1, the sample was similar to the most recent UK Census information in 2001 with regards to demographic and socioeconomic indicators for the catchment area under study, with the exception of this sample being slightly younger and having more students (25.8 percent versus 48.0 percent, not shown).

**Table 1:** Comparisons of SELCoH sample with available UK census information

	2001 UK Census for the SELCoH study catchment area^a^ n (%)	SELCoH study sample n (%)
**Total samples**^**b**^	N = 511035	N = 1698
**Gender**		
Female	260066 (50.9%)	959 (56.5%)
Male	250969 (49.1%)	739 (43.5%)
**Ethnic group**		
White	320377 (62.7%)	1051 (63.4%)
Mixed ^c^	22014 (4.3%)	---
Black-Caribbean	51694 (9.9%)	143 (8.7%)
Black-African	70186 (7.3%)	234 (13.2%)
Asian or Asian British	22105 (4.3%)	63 (3.5%)
Other	36593 (7.2%)	205 (11.2%)
**Age groups**		
16-29	129290 (32.6%)	577 (34.0%)
30-59	200387 (50.5%)	876 (51.6%)
60+	66770 (16.8%)	244 (14.4%)
**Economically active ^d^**	265546 (68.5%)	1125 (69.5%)
**Economically inactive^e^**	121919 (31.5%)	494 (30.5%)

^a^South east London Boroughs of Lambeth and Southwark; data are provided by the UK Office for National Statistics

^b^Census sample are age16 to 74 years and SELCoH sample are age 16 to 90; Frequencies may not add up to 100% due to missing values; percentages are unweighted

^c^Mixed ethnicity not specified as a category in the SELCoH study and are included in the Other ethnic category

^d^Economically active includes: Full time work, Part time work, Casual work, Unemployed, and Working Students

^e^Economically inactive includes: Student, Permanent sick/disabled, Temporary sick, Retired, Looking after the home children

### Common mental disorder

The mean total score for the CIS-R was 7.6 (SD 8.6, range 0-49) and CMD was present in 24.2% of the sample, 17.9% of men and 27.3% of women. As described in Table 2, CMD was associated with female gender, but not with age, borough or ethnicity. However, post hoc analysis showed that the Black Caribbean group had an increased likelihood of meeting the criteria for CMD in comparison to the Black African group [OR = 1.9, (C.I.= 1.1-3.2), p = 0.02, not shown]. In terms of relationship status, being in the married or cohabiting group had a decreased likelihood of CMD in comparison to the never married group. For socioeconomic indicators, there was an association between educational attainment at all levels in comparison to having a higher degree or above and CMD with evidence of a gradient across groups (p < 0.003 on one degree of freedom). CMD prevalence did not differ between non-manual and manual social class groups, but was associated with non-employed status, particularly in unemployed and temporary and permanent sick groups. There was also an association with lower reported household income and evidence of a gradient across categories (p < 0.001 on one degree of freedom) with a three-fold difference between the lowest and highest income groups. CMD was also higher in those living in rented accommodation compared to home owners or mortgage holders.

**Table 2:** Prevalence estimates and odds ratios (OR) for common mental disorder on the CIS-R (12+)

	n	N	Prevalence (95%CI)	p-value‡	Unadjusted OR (95%CI)	Test for trend
**Demographic Indicators**						
Total sample			24.2 (21.9-26.5)			
Gender						
Female	265	959	27.3 (24.3-30.2)	< 0.001	1.7 (1.4-2.2)***	
Male	131	739	17.9 (15.0-20.8)		1.0	
Ethnic group						
White British	250	1051	24.3 (21.5-27.3)	0.19	1.0	
Black-Caribbean	41	143	31.0 (22.3-39.8)		1.4 (0.9-2.2)	
Black-African	44	234	19.5 (13.8-25.1)		0.8 (0.5-1.1)	
Asian	14	63	24.9 (15.9-33.9)		1.0 (0.6-1.7)	
Other	46	205	23.0 (16.8-29.3)		0.9 (0.6-1.4)	
Age (years)						
16-24	84	356	25.1 (20.2-30.0)	0.13	1.0	
25-34	88	404	22.8 (18.4-27.2)		0.9 (0.6-1.3)	
35-44	77	336	24.3 (19.4-29.1)		0.9 (0.7-1.4)	
45-54	75	264	30.1 (24.2-35.9)		1.3 (0.9-1.9)	
55-64	41	163	25.4 (18.2-32.5)		1.0 (0.6-1.6)	
65+	31	175	18.3 (12.3-24.3)		0.7 (0.4-1.1)	
Relationship status						
Never married	172	678	26.5 (22.9-30.2)	< 0.01	1.0	
Married/cohabiting	153	786	19.8 (16.8-22.9)		0.7 (0.5-0.9)**	
Divorced/separated	57	181	32.2 (25.1-39.4)		1.3 (0.9-1.9)	
Widowed	14	53	27.1 (14.9-39.4)		1.0 (0.5-1.9)	
Borough						
Southwark	197	851	23.7 (20.5-26.9)	0.69	0.9 (0.7-1.2)	
Lambeth	199	847	24.6 (21.4-27.8)		1.0	
**Socioeconomic Indicators**						
Educational attainment						
No qualifications	61	228	25.7 (19.8-31.6)	< 0.01	1.5 (1.0-2.1)*	p = 0.003
Up to GCSE level	100	332	30.5 (25.2-35.8)		1.8 (1.3-2.5)***	
Advanced level	102	426	25.6 (21.2-29.9)		1.4 (1.1-1.9)*	
Higher degree or above	127	693	19.2 (16.1-22.3)		1.0	
Social class						
Non-manual	135	703	19.9 (16.7-23.1)	< 0.01	1.0	
Manual	51	244	22.2 (16.4-27.9)		1.1 (0.8-1.7)	
No current occupation	201	714	28.5 (24.9-32.1)		1.6 (1.2-2.1)***	
Employment status						
Full time	122	662	18.9 (15.8-22.2)	< 0.001	1.0	
Part time/casual	57	259	22.6 (17.2-28.1)		1.2 (0.9-1.8)	
Student/student working	46	247	19.8 (14.6-25.1)		1.1 (0.7-1.6)	
Unemployed	58	170	35.5 (27.9-43.0)		2.3 (1.6-3.4)***	
Temporary and permanent sick	54	81	67.0 (56.4-77.6)		8.7 (5.1-14.7)***	
Retired	36	188	19.8 (13.8-25.8)		1.1 (0.7-1.6)	
Home looking after children	21	82	25.2 (15.8-34.6)		1.4 (0.8-2.5)	
Yearly household income						
£0 - £5,475	60	139	42.2 (33.3-51.2)	< 0.001	3.2 (2.1-4.8)***	< 0.001
£5476 - £12,097	58	212	26.6 (20.5-32.7)		1.6 (1.1-2.3)*	
£12,098 - £20,753	56	203	28.9 (22.4-35.5)		1.8 (1.2-2.6)**	
£20,754 - £31,494	40	179	23.2 (15.8-30.6)		1.3 (0.8-2.0)	
£31,495 or more	129	703	18.8 (15.7-22.8)		1.0	
Housing tenure						
Own/mortgage	93	525	18.9 (15.1-22.7)	< 0.001	1.0	
Rented	286	1058	27.9 (24.8-30.9)		1.7 (1.2-2.2)***	
Rent free	16	112	14.2 (7.4-21.1)		0.7 (0.4-1.3)	

Weighted percentages to account for survey design; frequencies are unweighted and may not add up due to missing values.

‡Pearson's χ^2 ^test with Rao & Scott correction for survey data.

*p < 0.05; **p < 0.01; ***p < 0.001

### Hazardous alcohol consumption

Table 3 presents the prevalence estimates and factors associated with hazardous alcohol consumption. Women had a decreased likelihood of hazardous alcohol consumption in comparison to men and risk of hazardous alcohol consumption decreased with age. A test for a gender by age interaction was not significant at the 5% level (data available upon request). In comparison to the White British group, all ethnic groups with the exception of the Asian group were associated with reduced odds of hazardous alcohol consumption. All relationship status groups were associated with decreased odds of hazardous alcohol consumption in comparison to the never married group. Among the socioeconomic indicators, lower educational attainment, manual social class, economically inactive employment status groups and lower household income decreased odds of hazardous alcohol consumption. Alcohol consumption did not differ by housing tenure or borough of residence.

**Table 3:** Prevalence estimates and odds ratios (OR) for hazardous alcohol use (≥8 on the AUDIT)

	n	N	Prevalence (95%CI)	p-value‡	Unadjusted OR (95%CI)
**Demographic Indicators**					
Total sample			15.7 (13.8-17.6)		
Gender					
Female	120	959	11.3 (9.2-13.3)	< 0.001	0.4 (0.3-0.5)***
Male	193	739	24.6 (21.2-27.9)		1.0
Ethnic group					
White British	261	1051	20.5 (17.9-23.2)	< 0.001	1.0
Black-Caribbean	6	143	3.8 (0.8-6.8)		0.2 (0.1-0.4)***
Black-African	9	234	3.4 (1.2-5.7)		0.1 (0.1-0.3)***
Asian	11	63	16.3 (6.9-25.6)		0.8 (0.4-1.5)
Other	26	205	11.8 (7.2-16.4)		0.5 (0.3-0.8)***
Age (years)					
16-24	83	356	22.7 (17.8-27.7)	< 0.001	1.0
25-34	98	404	22.7 (18.3-27.1)		1.0 (0.7-1.4)
35-44	72	336	19.5 (15.1-23.9)		0.8 (0.6-1.2)
45-54	33	264	11.2 (7.3-15.1)		0.4 (0.3-0.7)**
55-64	20	163	10.2 (5.6-14.8)		0.4 (0.2-0.7)**
65+	7	175	3.3 (0.8-5.7)		0.1 (0.1-0.3)***
Relationship status					
Never married	159	678	21.4 (17.9-24.9)	0.001	1.0
Married/cohabiting	121	786	12.9 (10.5-15.3)		0.5 (0.4-0.7)***
Divorced/separated	30	181	14.1 (9.2-19.0)		0.6 (0.4-0.9)*
Widowed	3	53	4.8 (0.7-10.2)		0.2 (0.5-0.6)**
Borough					
Southwark	150	851	16.3 (13.6-19.0)	0.53	0.9 (0.7-1.2)
Lambeth	163	847	15.1 (12.4-17.8)		1.0
**Socioeconomic Indicators**					
Educational attainment					
No qualifications	28	228	9.9 (5.9-13.9)	< 0.001	0.4 (0.2-0.6)***
Up to GCSE level	38	332	9.3 (6.4-12.2)		0.4 (0.3-0.5)***
Advanced level	73	426	15.6 (11.9-19.3)		0.7 (0.5-0.9)**
Higher degree or above	172	693	21.7 (18.5-24.9)		1.0
Social class					
Non-manual	156	703	19.5 (16.5-22.6)	0.001	1.0
Manual	28	244	9.8 (6.0-13.6)		0.4 (0.3-0.7)**
No current occupation	119	714	13.9 (11.2-16.7)		0.7 (0.5-0.9)**
Employment status					
Full time	157	662	21.0 (17.7-24.3)	< 0.001	1.0
Part time/casual	25	259	8.2 (5.0-11.4)		0.3 (0.2-0.5)***
Student/student working	56	247	22.5 (16.1-28.8)		1.1 (0.7-1.6)
Unemployed	36	170	20.6 (14.2-27.0)		0.9 (0.6-1.5)
Temporary and permanent sick	17	81	16.7 (8.1-25.2)		0.8 (0.4-1.4)
Retired	16	188	6.5 (3.3-9.8)		0.3 (0.1-0.5)***
Home looking after children	5	82	6.3 (0.9-11.7)		0.3 (0.1-0.6)**
Yearly household income					
£0 - £5,475	32	139	18.9 (12.5-25.3)	< 0.001	0.8 (0.5-1.2)
£5476 - £12,097	26	212	10.4 (6.5-14.4)		0.4 (0.2-0.6)***
£12,098 - £20,753	16	203	6.5 (3.2-9.8)		0.2 (0.1-0.4)***
£20,754 - £31,494	29	179	14.7 (9.5-19.8)		0.6 (0.4-0.9)**
£31,495 or more	182	703	22.9 (19.6-26.2)		1.0
Housing tenure Own/mortgage	88	525	13.9 (10.9-16.9)	0.36	1.0
Rented	203	1058	16.4 (13.9-18.9)		1.2 (0.9-1.7)
Rent free	22	112	18.3 (10.1-26.5)		1.4 (0.8-2.5)

Weighted percentages to account for survey design; frequencies are unweighted and may not add up due to missing values.

‡Pearson's χ^2 ^test with Rao & Scott correction for survey data.

*p < 0.05; **p < 0.01; ***p < 0.001

### Self-rated health

Table 4 summarises the prevalence of and factors associated with fair or poor general health. Women, those in the Black Caribbean group, those who are in age groups 45 years and older, and those who reported being divorced or separated had increased odds of reporting fair or poor general health. Borough of residence was not associated with this outcome. Lower educational attainment, unemployment and being economically inactive, lower household income and living in rented accommodation increased the odds of reporting fair or poor health. Notably, those with no qualifications had a four-fold increase in the odds of reporting fair or poor general health in comparison to the reference group and there was evidence of a gradient across categories (p < 0.001, on one degree of freedom). There was also evidence of a gradient across income groups (p < 0.01, on one degree of freedom). There was no difference between non-manual and manual social class group in terms of odds of fair or poor general health.

**Table 4:** Prevalence estimates and odds ratios (OR) for fair or poor general health

	n	N	Prevalence (95%CI)	p-value‡	Unadjusted OR (95%CI)	Test for trend
**Demographic Indicators**						
Total sample			19.2 (17.0-21.3)			
Gender						
Female	185	954	21.5 (17.7-23.3)	0.05	1.3 (1.0-1.7)*	
Male	111	734	16.5 (13.6-19.4)		1.0	
Ethnic group						
White British	172	1049	17.6 (15.0-20.3)	0.01	1.00	
Black-Caribbean	39	142	29.9 (21.6-38.3)		2.0 (1.3-3.1)**	
Black-African	31	231	14.6 (9.5-19.6)		0.8 (0.5-1.2)	
Asian	11	62	22.3 (8.6-36.1)		1.3 (0.6-3.0)	
Other	42	202	23.6 (17.1-30.1)		1.4 (0.9-2.1)	
Age (years)						
16-24	48	356	14.1 (10.3-17.9)	< 0.001	1.0	
25-34	41	404	10.3 (7.2-13.3)		0.7 (0.4-1.1)	
35-44	50	336	15.3 (11.2-19.5)		1.1 (0.7-1.7)	
45-54	69	260	27.7 (22.1-33.4)		2.3 (1.6-3.5)***	
55-64	42	159	26.1 (18.6-33.6)		2.2 (1.3-3.6)**	
65+	46	173	26.8 (20.1-33.6)		2.2 (1.4-3.6)***	
Relationship status						
Never married	111	677	17.3 (14.2-20.4)	< 0.001	1.0	
Married/cohabiting	120	779	16.8 (13.8-19.7)		0.9 (0.7-1.3)	
Divorced/separated	51	179	30.1 (22.9-37.3)		2.1 (1.4-3.1)***	
Widowed	14	53	27.1 (14.8-39.4)		1.8 (0.9-3.4)	
Borough						
Southwark	157	844	17.9 (15.0-20.9)	0.26	1.2 (0.9-1.6)	
Lambeth	139	844	20.4 (17.2-23.6)		1.0	
**Socioeconomic Indicators**						
Educational attainment						
No qualifications	81	222	35.8 (28.9-42.6)	< 0.001	4.8 (3.3-7.2) ***	< 0.001
Up to GCSE level	78	330	25.7 (20.4-30.9)		3.0 (2.1-4.4) ***	
Advanced level	62	424	15.5 (11.7-19.2)		1.6 (1.1-2.4)*	
Higher degree or above	67	693	10.3 (7.9-12.7)		1.0	
Social class						
Non-manual	71	703	10.3 (7.9-12.7)	< 0.001	1.0	
Manual	33	244	14.4 (9.6-19.1)		1.5 (0.9-2.3)	
No current occupation	186	704	28.4 (24.6-32.1)		3.4 (2.5-4.7) ***	
Employment status						
Full time	72	662	11.4 (8.8-14.1)	< 0.001	1.0	
Part time/casual	30	259	11.4 (7.4-15.3)		1.0 (0.6-1.6)	
Student/student working	26	247	10.8 (6.9-14.7)		0.9 (0.6-1.5)	
Unemployed	42	169	26.3 (19.1-33.5)		2.8 (1.8-4.3)***	
Temporary and permanent sick	59	78	78.0 (68.7-87.3)		27.6 (15.0-50.5)***	
Retired	54	183	29.1 (22.4-35.8)		3.2 (2.1-4.9)***	
Home looking after children	12	81	15.1 (7.2-22.9)		1.4 (0.7-2.7)	
Yearly household income						
£0 - £5,475	54	137	40.7 (31.7-49.7)	< 0.001	6.5 (4.1-10.2)***	< 0.001
£5476 - £12,097	53	210	26.1 (19.8-32.4)		3.3 (2.2-5.1)***	
£12,098 - £20,753	49	203	25.9 (19.3-32.6)		3.3 (2.2-5.1)***	
£20,754 - £31,494	31	177	17.6 (11.1-24.1)		2.0 (1.2-3.4)**	
£31,495 or more	64	703	9.6 (7.2-11.8)		1.0	
Housing tenure						
Own/mortgage	54	523	11.1 (8.1-14.1)	< 0.001	1.0	
Rented	225	1051	23.8 (20.8-26.7)		2.5 (1.8-3.5)***	
Rent free	17	111	16.1 (8.8-23.3)		1.5 (0.8-2.9)	

Weighted percentages to account for survey design; frequencies are unweighted and may not add up due to missing values.

‡Pearson's χ^2 ^test with Rao & Scott correction for survey data.

*p < 0.05; **p < 0.01; ***p < 0.001

### Long standing illness

Table 5 shows the prevalence estimates and factors associated with having a long standing illness. There was no association between gender and having a long standing illness. In comparison to the White British ethnic group, those in the Black African group had reduced odds of having a long standing illness. There was a graded increase in the odds of long standing illness across age categories 35 years and older in comparison to the youngest age group, and all relationship status groups were associated with increased odds of having a long standing illness in comparison to the never married group. Lower educational attainment groups, those in the economically inactive groups, and lower household income were associated with increased odds of having a long standing illness.

**Table 5:** Prevalence estimates and odds ratios (OR) for having a long standing illness

	n	N	Prevalence (95%CI)	p-value‡	Unadjusted OR (95%CI)
**Demographic Indicators**					
Total sample			44.9 (42.4-47.6)		
Gender					
Female	394	953	46.1 (42.8-49.4)	0.17	1.1 (0.9-1.4)
Male	279	735	42.8 (39.0-46.5)		1.0
Ethnic group					
White British	439	1048	47.2 (43.9-50.5)	0.05	1.0
Black-Caribbean	56	142	44.9 (36.7-53.3)		0.9 (0.6-1.3)
Black-African	73	230	35.2 (28.3-42.0)		0.6 (0.4-0.8)**
Asian	24	63	42.9 (28.9-56.8)		0.8 (0.5-1.5)
Other	80	203	44.4 (37.0-51.8)		0.9 (0.6-1.2)
Age (years)					
16-24	80	356	23.2 (19.1-27.3)	< 0.001	1.0
25-34	83	402	20.6 (16.4-24.7)		0.9 (0.6-1.2)
35-44	123	336	37.4 (32.0-42.9)		1.9 (1.4-2.8)***
45-54	138	259	54.3 (48.1-60.6)		3.9 (2.8-5.6)***
55-64	109	161	69.0 (61.7-76.3)		7.4 (4.9-11.1)***
65+	140	174	79.9 (73.9-85.9)		13.1 (8.5-20.3)***
Relationship status					
Never married	212	674	34.2 (30.4-37.9)	< 0.001	1.0
Married/cohabiting	320	782	45.6 (41.7-49.5)		1.6 (1.3-2.0)***
Divorced/separated	103	179	61.1 (53.8-68.5)		3.0 (2.1-4.3)***
Widowed	38	53	73.6 (61.7-85.6)		5.4 (2.8-10.2)***
Borough					
Southwark	360	845	41.6 (38.0-45.2)	0.01	1.3 (1.1-1.6)**
Lambeth	313	843	48.3 (44.6-52.0)		1.0
**Socioeconomic Indicators**					
Educational attainment					
No qualifications	146	224	70.0 (64.0-76.0)	< 0.001	4.3 (3.1-5.9) ***
Up to GCSE level	144	329	50.2 (44.6-55.9)		1.9 (1.4-2.5) ***
Advanced level	146	426	36.8 (31.9-41.6)		1.1 (0.8-1.4)
Higher degree or above	222	690	35.2 (31.5-38.9)		1.0
Social class					
Non-manual	217	700	33.1 (29.4-36.9)	< 0.001	1.0
Manual	77	244	35.9 (29.4-42.6)		1.1 (0.8-1.6)
No current occupation	362	708	57.2 (53.2-61.1)		2.7 (2.1-3.4) ***
Employment status					
Full time	202	658	33.2 (29.3-37.1)	< 0.001	1.0
Part time/casual	90	259	38.9 (32.6-45.4)		1.3 (0.9-1.8)
Student/student working	59	247	24.0 (18.8-29.2)		0.6 (0.5-0.9)**
Unemployed	65	168	40.5 (32.8-48.3)		1.4 (0.9-2.0)
Temporary and permanent sick	72	78	93.8 (88.8-98.8)		30.5 (12.8-72.9)***
Retired	151	187	80.6 (74.8-86.4)		8.4 (5.5-12.7)***
Home looking after children	31	82	39.8 (29.1-50.6)		1.3 (0.8-2.2)
Yearly household income					
£0 - £5,475	76	137	59.7 (51.7-67.8)	< 0.001	2.9 (2.1-4.3)***
£5476 - £12,097	118	210	62.8 (55.9-69.6)		3.4 (2.4-4.8)***
£12,098 - £20,753	91	203	49.9 (42.7-57.1)		2.0 (1.4-2.8)***
£20,754 - £31,494	63	178	41.2 (33.4-49.1)		1.4 (0.9-2.0)
£31,495 or more	218	701	33.2 (29.5-36.9)		1.0
Housing tenure					
Own/mortgage	217	525	44.9 (40.5-49.5)	< 0.001	1.0
Rented	433	1052	47.3 (43.9-50.6)		1.1 (0.9-1.4)
Rent free	23	109	21.1 (13.1-29.2)		0.3 (0.2-0.5)***

Weighted percentages to account for survey design; frequencies are unweighted and may not add up due to missing values.

‡Pearson's χ^2 ^test with Rao & Scott correction for survey data.

*p < 0.05; **p < 0.01; ***p < 0.001

### Comparisons across health outcomes

In fully adjusted models in Table 6, women were at increased risk for CMD, men for hazardous alcohol use and where ethnicity was associated with health outcomes, those in ethnic minority groups had decreased odds of poor health outcomes. The odds of hazardous alcohol use decreased with age and were reduced for those in the married or cohabitating group. Among the socioeconomic indicators, higher educational attainment and higher household income increased the odds of hazardous alcohol use. Although some associations between socioeconomic indicators and health outcomes were not present in the fully adjusted model, the direction of these associations for hazardous alcohol consumption was opposite to all other health outcomes.

**Table 6:** Odds ratios (OR) for all health outcomes in fully adjusted models^a^

	Common mental disorder	Hazardous alcohol use	Fair or poor general health	Long standing illness
	**Adjusted OR (95%CI)**	**Adjusted OR (95%CI)**	**Adjusted OR (95%CI)**	**Adjusted OR (95%CI)**

**Demographic Indicators**				
Gender				
Female	1.4 (1.1-1.9)*	0.3 (0.2-0.4)***	1.2 (0.8-1.6)	1.1 (0.9-1.4)
Male	1.0	1.0	1.0	1.0
Ethnic group				
White British	1.0	1.0	1.0	1.0
Black-Caribbean	0.9 (0.6-1.7)	0.2 (0.1-0.5)***	1.4 (0.8-2.4)	0.7 (0.4-1.2)
Black-African	0.6 (0.4-0.9)*	0.1 (0.1-0.3)***	0.7 (0.4-1.1)	0.8 (0.6-1.2)
Asian	0.9 (0.5-2.0)	1.0 (0.5-2.1)	1.7 (0.7-4.0)	1.1 (0.6-2.1)
Other	0.8 (0.5-1.1)	0.4 (0.2-0.7)**	1.4 (0.9-2.2)	1.1 (0.8-1.6)
Age (continuous in years)	1.0 (0.9-1.0)	0.9 (0.9-0.9)***	1.0 (0.9-1.0)	1.1 (1.0-1.1)***
Relationship status				
Never married	1.0	1.0	1.0	1.0
Married/cohabiting	0.8 (0.6-1.1)	0.6 (0.4-0.9)**	0.9 (0.6-1.3)	0.8 (0.6-1.1)
Divorced/separated	1.1 (0.7-1.8)	1.3 (0.7-2.3)	1.3 (0.8-2.2)	0.9 (0.6-1.5)
Widowed	1.1 (0.5-2.4)	0.9 (0.2-3.7)	0.5 (0.2-1.3)	0.4 (0.2-1.0)
**Socioeconomic Indicators**				
Educational attainment				
No qualifications	1.4 (0.8-2.3)	1.0 (0.5-1.9)	1.7 (0.9-3.1)	1.0 (0.6-1.7)
Up to GCSE level	1.5 (0.9-2.2)	0.5 (0.3-0.8)**	1.6 (0.9-2.6)	1.2 (0.8-1.7)
Advanced level	1.2 (0.8-1.7)	0.7 (0.5-0.9)*	1.0 (0.6-1.6)	1.0 (0.7-1.4)
Higher degree or above	1.0	1.0	1.0	1.0
Employment status				
In paid employment	1.0	1.0	1.0	1.0
Unemployed	1.3 (0.8-2.1)	1.7 (0.9-3.0)	1.6 (0.9-2.7)	1.0 (0.6-1.7)
Economically inactive	1.1 (0.8-1.6)	1.2 (0.8-1.7)	1.7 (1.1-2.5)**	1.7 (1.2-2.3)**
Yearly household income				
£0 - £5,475	2.2 (1.3-3.7)**	1.0 (0.6-1.9)	2.5 (1.4-4.6)**	1.7 (1.0-2.8)*
£5476 - £12,097	1.2 (0.7-1.9)	0.6 (0.3-0.9)*	1.2 (0.7-2.2)	1.5 (0.9-2.3)
£12,098 - £20,753	1.4 (0.9-2.1)	0.3 (0.2-0.6)***	1.6 (0.9-2.7)	1.2 (0.8-1.7)
£20,754 - £31,494	1.1 (0.7-1.8)	0.7 (0.4-1.2)	1.4 (0.8-2.5)	1.2 (0.8-1.8)
£31,495 or more	1.0	1.0	1.0	1.0
Housing tenure				
Own/mortgage	1.0	1.0	1.0	1.0
Rented	1.3 (0.9-1.9)	1.3 (0.9-1.9)	1.9 (1.3-3.0)**	1.3 (0.9-1.9)
Rent free	0.3 (0.1-0.7)**	0.9 (0.4-1.9)	1.4 (0.6-3.6)	0.7 (0.3-1.7)

^a ^Models contain all demographic and socioeconomic indicators.

Economically inactive includes: student, permanent sick/disabled, temporary sick, retired, looking after the home children

*p < 0.05; **p < 0.01; ***p < 0.001

### Relationship between health outcomes

The co-occurrence of health outcomes (Table 7) showed that there was no association between hazardous alcohol use and fair or poor health or with reports of longstanding illness. In comparison, those who reported hazardous alcohol use and having a longstanding illness were both two times more likely to meet the criteria for CMD. There were also strong associations between reporting fair or poor health and CMD, as well as fair or poor health and longstanding illness.

**Table 7:** Relationship between all health outcomes

	Common mental disorder	Hazardous alcohol use	Fair or poor general health
**Health Indicators**	Adjusted OR (95%CI)	Adjusted OR (95%CI)	Adjusted OR(95%CI)
Hazardous alcohol use			
Yes	2.0 (1.4-2.9)***		
No	1.0		
Fair or poor general health			
Yes	6.1 (4.3-8.5)***	1.3 (0.9-2.1)	
No	1.0	1.0	
Long standing illness			
Yes	2.4 (1.8-3.2)***	1.3 (0.9-1.8)	5.3 (3.8-7.4)***
No	1.0	1.0	1.0

Models are adjusted for gender, age (continuous), ethnicity, relationship status, employment status, household income and housing tenure

*p < 0.05; **p < 0.01; ***p < 0.001

### Health indicators and functioning

In comparisons to those who reported no limits to social functioning, all poor health indicators were associated with increased risk of reporting limitations to social functioning (Table 8). With the exception of hazardous alcohol consumption, poor mental and physical health indicators were associated with functional limitations due to both physical and emotional health. Hazardous alcohol consumption was associated with functional limitations as a result of emotional health, but was not associated with functional limitations due to physical health.

**Table 8:** Prevalence estimates and associations for health and functioning

		Limitations for social functioning				
	Prevalence (95%CI)		Unadjusted RRR^†† ^(95%CI)		Adjusted RRR^††† ^(95%CI)	
**Health Indicators**	Most or all of the time	Some of the time	Most or all of the time	Some of the time	Most or all of the time	Some of the time

Common mental disorder						
Yes	25.2 (20.4-29.9)	48.9 (43.8-54.2)	27.5 (17.1-44.4)***	7.5 (5.7-9.9)***	22.3 (13.0-38.2)***	6.9 (5.0-9.4)***
No	2.7 (1.8-3.7)	19.5 (17.2-21.9)				
Hazardous alcohol use						
Yes	8.0 (4.6-11.4)	34.0 (28.2-39.8)	1.2 (0.7-1.9)	1.6 (1.2-2.1)**	2.3 (1.2-4.4)**	1.4 (1.0-1.9)*
No	7.9 (6.3-9.5)	25.1 (22.7-27.6)				
Fair or poor general health						
Yes	25.4 (19.9-30.9)	37.5 (31.5-43.4)	12.2 (7.9-18.8)***	3.0 (2.2-4.1)***	8.4 (5.2-13.6)***	3.2 (2.2-4.5)***
No	4.0 (2.9-5.1)	24.0 (21.5-26.4)				
Long standing illness						
Yes	13.8 (11.0-16.6)	28.6 (24.9-32.2)	5.0 (3.3-7.7)***	1.4 (1.1-1.8)**	3.8 (2.3-6.4)***	1.8 (1.3-2.4)***
No	3.4 (2.2-4.6)	24.9 (22.0-27.8)				

		**Physical health limits functioning **				
**Health Indicators**	Prevalence (95%CI)		Unadjusted OR (95%CI)	Adjusted^† ^OR (95%CI)		

Common mental disorder						
Yes	45.2 (39.9-50.4)		4.9 (3.7-6.4)***	5.3 (3.8-7.2)***		
No	14.5 (12.4-16.7)		1.0	1.0		
Hazardous alcohol use						
Yes	18.7 (13.8-23.6)		0.8 (0.6-1.1)	1.2 (0.8-1.8)		
No	22.3 (19.8-24.9)		1.0	1.0		
Fair or poor general health						
Yes	56.3 (50.2-62.4)		8.2 (6.1-10.9)***	6.6 (4.7-9.2)***		
No	13.6 (11.7-15.6)		1.0	1.0		
Long standing illness						
Yes	38.8 (34.9-42.8)		7.2 (5.4-9.7)***	4.9 (3.5-6.9)***		
No	8.1 (6.3-9.9)		1.0	1.0		

		**Emotional health limits functioning**				
**Health Indicators**	Prevalence (95%CI)		Unadjusted OR (95%CI)	Adjusted^† ^OR (95%CI)		

Common mental disorder						
Yes	50.6 (45.3-55.9)		17.7 (12.7-24.8)***	15.4 (10.6-22.5)***		
No	5.5 (4.1-6.8)		1.0	1.0		
Hazardous alcohol use						
Yes	21.4 (16.4-26.3)		1.5 (1.1-2.1)*	1.7 (1.2-2.5)**		
No	15.3 (13.2-17.3)		1.0	1.0		
Fair or poor general health						
Yes	35.5 (29.6-41.4)		4.1 (3.0-5.7)***	3.7 (2.6-5.2)***		
No	11.7 (9.9-13.5)		1.0	1.0		
Long standing illness						
Yes	21.6 (18.3-24.9)		2.0 (1.5-2.7)***	2.2 (1.6-3.1)***		
No	11.9 (9.9-14.0)		1.0	1.0		

Weighted percentages to account for survey design; frequencies are unweighted and may not add up due to missing values.

^†^Adjusted for gender, age (continuous), ethnicity, relationship status, employment status, household income and housing tenure; economically inactive includes: student, permanent sick/disabled, temporary sick, retired, looking after the home children

^††^Relative risk ratios derived from multinomial (polytomous) logistic regression; social functioning limited none of the time is the reference group

*p < 0.05; **p < 0.01; ***p < 0.001

## Discussion

Using data from the SELCoH study, an epidemiological study of an inner city community population, we aimed to identify the demographic and socioeconomic distribution of common mental disorder (CMD), hazardous drinking, long standing illness and self-rated health. Our findings identified high risk groups across health outcomes and there were notable differences between hazardous alcohol use and the other health outcomes. The prevalence of poor health outcomes by ethnic group suggests that there are important differences between groups. In particular, patterns for hazardous alcohol use highlight a risk group characterised by being male, younger, never married, identifying their ethnicity as White and being in higher SES categories. Women and individuals who are socioeconomically disadvantaged were worse off for other outcomes. Overall, there is considerable co-occurrence of poor mental and physical problems and this is likely to contribute to the extent of functional limitations resulting from health problems, particularly in the presence of poor mental health.

Several of the findings are consistent with previous studies at the community and national levels. As in many previous national studies [40-42], our findings indicate that there is a continued need to address public mental health issues among women with regards to internalising symptoms, such as anxiety and depression captured in measures of CMD. However, the gender effects are distinct from the impact of low SES on CMD; thus, interventions addressing CMD in the community should also benefit men and those in low SES groups. Previous findings have shown an increase in reporting of symptoms in young adulthood that decreases with increasing age [40,43]. Thus, it was somewhat unexpected that there was no difference in the prevalence of CMD across most age groups, with the exception of the oldest, age 65 years and older. Further, the overall findings for comparisons across all ethnic groups are consistent with previous studies of CMD at the national level group [44,45], despite having sufficient proportions of members from ethnic minority groups.

In terms of the proportion of individuals with poor health outcomes in local community samples, this population fares worse in terms of self-rated health in comparison to samples from epidemiological community samples with similar design in urban areas, such as the Baltimore Epidemiological Catchment Area study in the US [46]. Interestingly, those who identified themselves as being in the Black Caribbean group had markedly poorer health than those who identified as being in the Black African group on all health indicators except hazardous alcohol use. While there was no difference across all ethnic groups for CMD, post hoc analysis showed that the Black Caribbean group were at increased risk for common mental disorder in comparison to the Black African group. This is especially notable given that these two groups have been combined in a large number of health studies in the UK because of small group sample sizes. It also suggests that making comparisons between broad ethnic groups (e.g. white versus black) may lead to flawed inferences. As in other community studies with large proportions of migrants (39.5% in our sample), this could represent what has been termed the 'healthy migrant effect' (i.e., migrants who have recently arrived have better health than the native population, but their health deteriorates after [5]); however, while 77.5% in the Black African group were born outside of the UK, the overwhelming majority have lived in the UK for over ten years at the time of the present study. Future analysis needs to consider other social factors previously identified as being protective, such as social integration and support. Because the Black population is approximately a quarter of the total population in the SELCoH study sample (and the target population according to the last UK census), these health differences are particularly informative for local health providers and policy makers.

For all health outcomes, the evidence suggests that there are similar patterns for individual- and household-level SES indicators (i.e., education, occupational social class, income, employment status and housing tenure); however the traditional use of occupational social class is not as informative as educational level, income and occupational status. Our findings for increased fair or poor general health among groups that identified as Black Caribbean, in the other ethnic category, never married or in low SES groups require more attention. It is possible that other social exclusion factors are contributing to this health assessment that has been so strongly correlated to increased morbidity and mortality [20]. There is also a need to disaggregate the impact of individual and household SES factors [47].

These findings suggest that routinely capturing these SES indicators during health visits could alert health practitioners to identifying groups that may be particularly at differential high risk for various poor health outcomes, though not necessarily in a predictable manner. For example, identifying someone with high SES group in this community should signal the need to inquire about alcohol intake. Given that these findings are not consistent with the finding showing no differences by SES in the 2000 National Psychiatric Morbidity survey in the UK [48], these demographic differences in health at the local level should continue to direct local public health policies and messages. For example, public health policies, such as raising the price of alcohol to tackle binge drinking [49,50], may have an influence on young adult alcohol consumptions but are less likely to have an impact on high SES groups. There is also a need to consider the high risk groups defined by multiple socio-demographic indicators. With regards to gender and age, consistent with previous national studies, our findings suggest that men [48,51,52] and young adults [52,43] should be considered alongside those in high SES groups for hazardous alcohol consumption.

Finally, the relationship between the health outcomes and the relationship between the health outcomes and functioning portrayed the complexity of ill health for most people. Our findings suggest that the co-occurrence of mental and physical ill health is common in the local population, with particular implications for functioning. Nearly half of the participants with CMD reported having a long standing illness, such as high blood pressure and asthma, and the proportion of those with CMD with functional limitations were similar or greater to those with poor physical health. In addition, as in previous studies, we demonstrated that self-rated health is an important indicator of the multiple dimensions of health [21]. However, self-ratings of health do not relate to hazardous alcohol use in our sample. The findings do suggest that the relationship between hazardous alcohol use and CMD deserve further attention.

### Strengths and limitations

This study administered a validated structured psychiatric interview (CIS-R) and additional health assessments on a diverse sample residing in an urban community setting with high levels of social deprivation. In considering mental health as an outcome, the focus of these analyses was on the generic category of 'common mental disorder'. Differentiation between component diagnostic groups was beyond the scope here but will be considered in future output. We acknowledge concerns about the validity of measures, such as the CIS-R being administered by trained lay interviewers [53]. However, as with all major mental health population surveys, we enlisted experienced and trained lay interviewers to administer the CIS-R. The interviewers successfully undertook extensive fieldwork to access individuals residing in private households in one of the largest and most diverse cities in the world. The decline in the number of community epidemiological studies of this nature, in part, reflects how difficult these studies are to complete. The 51.9% household participation rate indicates that participation bias is likely and prevalence estimates should be considered with caution. Despite this, the household participation rate and the 71.9% participation rate among eligible household members, taken together, were relatively high given the level of deprivation in the area. Further, a recent simulation study illustrated that nonparticipation may be less influential in studies of associations between exposures and outcomes [54]. Generalizability may be limited since this study took place in two of the boroughs in south east London and there were insufficient participants from South Asian populations (Indian, Pakistani, Bangladeshi) to consider these groups separately. Further, the cross-sectional study design limits our ability to make causal inferences or go beyond a theoretical discussion about these demographic and socioeconomic factors as determinants of health. However, these limitations do not detract from the rich, descriptive data on a sample that closely reflects the demographic make up of the population from which it was drawn and inform public health needs of this and similar populations.

## Conclusion

The present survey was, in part, a response to the needs of the local health economy to develop a public health strategy in relation to the epidemiological information on the basic and relevant demographic and socioeconomic distribution of common mental disorders and general physical health in the local population. While identifying the prevalence and distribution of health inequalities by demographic and socioeconomic factors is particularly important at the local community level, it does not detect the proportion of people that are actually in need of treatment or how health services can meet the challenge of such a large proportion of people, particularly those with co-occurring mental and physical ill health. The greatest challenge to functioning in daily life and for local health services will continue to be the presentation of mental ill health in combination with poor physical health. Population approaches to better health-related functioning and quality of life should consider this at every stage of improving care.

## Competing interests

The authors declare that they have no competing interests.

## Authors' contributions

MH designed the study, sought and obtained funding, oversaw data collection and analysis, and commented on drafts of the paper. SLH oversaw the data collection, wrote all drafts of the manuscript and conducted the analysis. SF and MV coordinated the data collection and commented on drafts of the paper. BK, JC, BG, RM and research assistants on the SELCoH team collected the data, prepared some of the descriptive analyses and commented on drafts of the paper. SLH, RS, NTF, CM and AR all contributed to the study's design and RS, NTF, CM and AR commented on drafts of the paper.

## Pre-publication history

The pre-publication history for this paper can be accessed here:

http://www.biomedcentral.com/1471-2458/11/861/prepub
